# Foot Posture Index Reference Values among Young Adults in Saudi Arabia and Their Association with Anthropometric Determinants, Balance, Functional Mobility, and Hypermobility

**DOI:** 10.1155/2021/8844356

**Published:** 2021-03-28

**Authors:** Khalid A. Alahmari, Venkata Nagaraj Kakaraparthi, Ravi Shankar Reddy, Paul Silvian Samuel, Jaya Shanker Tedla, Kanagaraj Rengaramanujam, Irshad Ahmad, Devika Rani Sangadala, Debjani Mukherjee

**Affiliations:** Department of Medical Rehabilitation Sciences, College of Applied Medical Sciences, King Khalid University, Abha, Saudi Arabia

## Abstract

**Background:**

The foot posture index (FPI) is a valid, reliable, and multidimensional method for determining foot posture in a wide range of clinical settings. To date, no normative data of healthy young adults in Saudi Arabia have been available for comparison and reference. Hence, this study is aimed at establishing the FPI reference values, gender, and side differences of FPI and their association with anthropometric determinants, balance, functional mobility, and hypermobility.

**Methods:**

FPI was assessed in 581 (291 men and 290 women) healthy young adults aged 18–25 years. The FPI range was obtained for both feet as the sum of the scores (–2, –1, 0, 1, and 2) given to each criterion: (–1 to –12) supinated foot, (0 to +5) neutral foot, and (+6 to +12) pronated foot. The study furthermore assessed the balance using a near tandem balance test, functional mobility by stair ascent and descent test, and joint hypermobility via the Beighton scale.

**Results:**

The average FPI score was 2.76 ± 5.23 for all subjects, 2.98 ± 5.02 for men and 2.55 ± 5.43 for women. Neutral foot posture was most frequent in this study (52.9%). A higher proportion of women had pronated (21.0%) and supinated (11.7%) feet than men which were 16.8% and 10.3%, respectively. This study also confirmed that side differences were found to be significant (*p* value < 0.001), whereas gender differences were significant only in the normal, pronated, and supinated foot groups.

**Conclusion:**

The most common foot posture in both genders was ranged from neutral to slight pronation. We also found a correlation between balance with FPI in the supinated and hypersupinated foot groups, functional mobility with FPI of pronated and supinated foot groups, and joint hypermobility with FPI of the hyperpronated foot group.

## 1. Introduction

The foot has a multifaceted efficient role in locomotion because it primarily supports the weight of the body and plays a vital role during movement [1]. However, due to poor foot posture in activities of daily living, such as standing, walking, and running, the foot is more susceptible to everyday strains [2]. Poor foot posture and misalignment have been reported to result in increased musculoskeletal injuries [3], which include low back pain [4], ankle/foot injuries due to overuse [5], patellofemoral pain syndrome [6], and medial stress syndrome [7]. Therefore, the primary aspect of an appropriate clinical response involves early assessment and evaluation of the foot posture.

Various methods have been identified in the literature to evaluate standing foot posture. These approaches include radiography [8], footprint method [9], arch height index [10], navicular drop test [11], and foot posture index (FPI) [12]. Out of all these measurements, only FPI does not require any sophisticated equipment. FPI is a reliable and easy-to-use test for health professionals to compare normative values on different population groups [12].

However, the normative data of FPI values for the healthy adult population is available only for a few countries. Gijon-Nogueron et al. found that the FPI range for healthy young adults in Spain goes from one point of supination to positive pronation [13]. Redmond et al. reported smaller positive FPI scores that typically represent the normal foot for young adults in the UK and Australia [14]. Cornwall and McPoil reported that the typical foot posture for healthy adults in the U.S.A. is a neutral position with respect to the FPI index [15]. Because FPI shows variations for different population groups, we intended to evaluate the reference values of FPI for healthy adults in Saudi Arabia.

Some studies have also determined that factors such as age, gender, anthropometric determinants, functional mobility, and balance have an association with FPI [16–18]. Significant differences in foot morphology, such as increased width and length of the feet between men and women during the growth phase, and increased BMI were considered to be causative factors that affect the medial longitudinal arch (MLA) [19]. To maintain the upright position during balance, both central and peripheral mechanisms of the nervous system regularly interact to control the position of the body and the center of gravity over the base of support [20, 21]. However, to our knowledge, no study has evaluated the association of various degrees of foot posture with anthropometric determinants, mobility, and balance to find the size effect of the foot group and gender on these variables among healthy young adults in Saudi Arabia.

Hence, the objectives of the present study were to (1) evaluate the reference values of FPI among young adults in Saudi Arabia according to their gender and side of the body; (2) analyze the balance, functional mobility, and hypermobility in different foot groups between male and female subjects; and (3) clarify the association of FPI with anthropometric determinants and other variables among young adults between men and women. We hypothesized that the differences in FPI values might be observed with the gender and side of the individuals. In addition, we also hypothesized that there might be an association of FPI with anthropometric determinants, balance, functional mobility, and hypermobility regardless of the foot posture type.

## 2. Material and Methods

### 2.1. Methods

In this cross-sectional study, the data were collected from 581 healthy subjects (291 men and 290 women) in the Physiotherapy Outpatient Department, King Khalid University, Saudi Arabia. Sample size calculation was performed prior to the study using G∗POWER statistical software (version 3.1.9.4; Universität Kiel, Germany). The participants were randomly chosen from the student and faculty registry of the University by utilizing a systematic random sampling method. The sampling interval (K) was computed using the formula (K = N/Tsz). N is denoted as the total number of students and faculties and was 1162, and Tsz is the total sample size. So, every 2nd volunteer was selected from the registry. The current university where the study was conducted has more than 170 colleges with various specialties. We selected four colleges randomly, and participants were chosen by random sampling. All required permissions were obtained from these colleges for conducting the study. Before performing the evaluation, the examiner explained the procedure and purpose of this research to all participants.

The inclusion criteria were (1) age between 18 and 30 years, (2) asymptomatic feet, (3) no obvious joint deformities, (4) no peripheral neuropathy or sensory deficits, and (5) no history of orthotic use for lower extremities. The exclusion criteria were (1) acute or chronic orthopaedic conditions, (2) history of spine or lower-limb surgeries, (3) congenital abnormalities, (4) vestibular impairments, (5) leg-length discrepancy, (6) plantar fasciitis, (7) any other medical conditions that interfere with balance, (8) any signs of foot pain for six months, (9) any painful cutaneous conditions, and (10) any pregnant women.

All subjects included in this study signed informed, written consent. The Ethical Committee of King Khalid University approved the present study protocol (ECM #2019-60).

### 2.2. Procedures

Demographic data (age, gender) and anthropometric factors, which include height (m), weight (kg), and BMI (kg/m^2^), were evaluated for all participants with a standard procedure. After completing this procedure, the FPI measurement, balance (near tandem balance test), functional mobility (stair ascent and descent test), and hypermobility (Beighton scale) were assessed. The experimenter has more than sixteen years of a good level of experience in performing these clinical tests.

### 2.3. FPI Measurement

**Figure 1:**
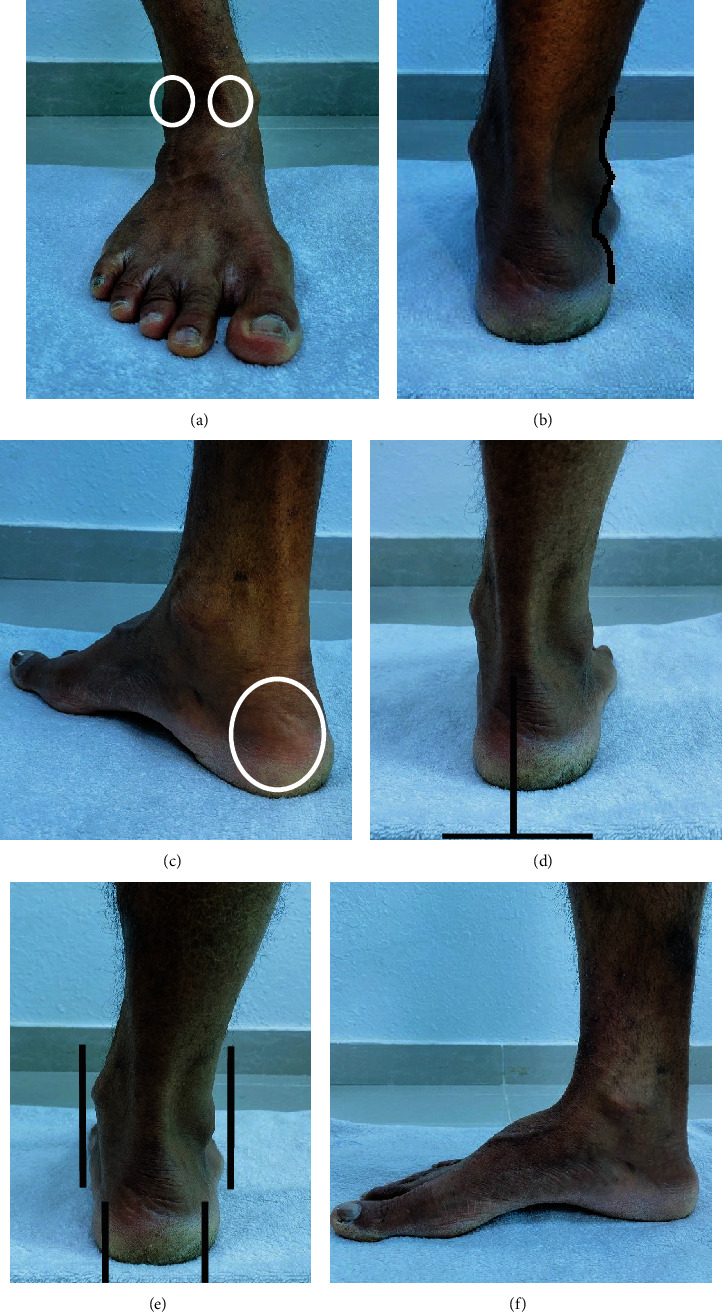
Foot posture index (FPI) measurement: (a) talar head position; (b) supra- and inframalleolar curvature; (c) talonavicular prominence; (d) calcaneal frontal plane position; (e) abduction/adduction of forefoot on rear foot; (f) medial longitudinal congruence.

All participants were asked to stand barefoot without any shoes or socks with double-limb support for the measurement of foot posture. Subjects were then instructed to take a few steps on the spot and stand in a relaxed stance position with upper extremities along with the trunk, with eyes looking forward, for approximately five to ten minutes. In this position, the individual foot was evaluated visually and palpated by the examiner. Six FPI criteria—(A) talar head palpation, (B) supra- and inframalleolar curvature, (C) talonavicular prominence, (D) calcaneal frontal plane position, (E) abduction or adduction of the forefoot on the rearfoot, and (F) medial longitudinal arch congruence—were evaluated (Figure 1). The scoring for each criterion followed a scale of –2, –1, 0, +1, or+2. Scores were added for each criterion. A score of –5 to –12 was labelled as a highly supinated foot, –1 to –4 as a supinated foot, 0 to +5 as a normal foot, +6 to +9 as a pronated foot, and +10 to +12 as a highly pronated foot [12]. All FPI values were taken by the same examiner to minimize measurement errors because the FPI had good intrarater reliability (ICC = 0.89–0.96) [12].

### 2.4. Near Tandem Balance Test

In this test, the subjects were asked to stand barefoot without any shoes or socks by placing one foot in front of another, laterally separated by 2.5 cm, with the front foot heel 2.5 cm anterior to the back foot great toe. The subjects chose which foot to place in the forward position for the test and stood in this position with their eyes closed. Scoring was based on the number of seconds the subjects maintained this position, and the scoring was stopped as soon as participants opened their eyes or when they took a step [18] (ICC = 0.94, SEM = 0.72) [22].

### 2.5. Stair Ascent and Descent Test

Participants were instructed to walk up and then down eight stairs barefoot without any shoes or socks as quickly as possible. The stairs were indoors, had a handrail, linoleum covered, and were well lit. Subjects were instructed to begin the test at the bottom of the eight stairs (15 cm high, 27.5 cm broad), and a stopwatch was used to record the performance. They were instructed to complete the task as quickly as possible, but not to run and to take only one step at a time. They were permitted to use the handrail if necessary. Timing was initiated for the stair ascent when the subjects raised their foot off the ground to climb the first step and stopped when both feet were positioned on the eighth step (a landing). After a short rest, subjects were asked to descend the stairs. The timing began when subjects raised their foot off the ground for the first step and stopped when the last step was completed. The total time taken to complete the test was recorded in seconds [23] for stair ascent and descent (ICC = 0.93, SEM = 0.098) [24].

### 2.6. Beighton Scale

This scale was used to check the presence of joint hypermobility on a 9-point scale at the wrist joint, fifth metacarpophalangeal joint, elbow joint, knee joint (all bilateral and non-weight-bearing), and spine. It mainly consists of a series of bilateral joint extensibility tests described as (1) passive dorsiflexion of the little fingers beyond 90° (one point for each hand), two points; (2) passive opposition of the thumb to the flexor aspects of the forearm (one point for each thumb), two points; (3) hyperextension of the elbow joint beyond 10° (one point for each elbow), two points; (4) hyperextension of the knee joint beyond 10° (one point for each knee), two points; and (5) forward flexion of the trunk with both knees fully extended so that the palms of the hands rest flat on the floor, one point [25]. Whether the tested joint was hypermobile (score = 1) or not hypermobile (score = 0) was entered into the evaluation sheet. Therefore, the total score fell between 0 and 9. A score of 5 or more out of 9 indicated hypermobility of the joints [26] (ICC = 0.72, SEM = 0.7) [27].

### 2.7. Data Analysis

SPSS software (version 21.0 for Windows; SPSS, Inc, Chicago, USA) was used to conduct statistical analyses. Descriptive statistics were used to characterize the sample. The Shapiro-Wilk test was conducted to check the normal distribution of data and homogeneity between groups. An independent Student *t*-test was used to evaluate the FPI values for gender and side of the foot. To compare the variances in balance, functional mobility, and hypermobility in all the foot groups among male and female subjects, univariate analysis of variance (ANOVA) was used, followed by post hoc Tukey. We also used Pearson correlation coefficients to assess the degree of relationship between the FPI values with age, height, weight, BMI, balance, functional mobility, and hypermobility. The significance level was set at a *p* value of <0.05 for all analyses.

## 3. Results

The anthropometric characteristics for all subjects are presented in Table 1, with a mean height of 1.70 ± 0.74 m for males and 1.57 ± 0.59 m for females, a mean weight of 73.99 ± 15.58 kg for males and 56.65 ± 9.16 kg for females, and mean BMI of 25.48 ± 5.17 kg/m^2^ for males and 22.92 ± 3.58 kg/m^2^ for females. The results showed that height, weight, and BMI between male and female subjects in both groups were statistically significant (*p* value < 0.001).

Of the 581 subjects (1162 feet) examined for both genders (men, *n* = 291 and women, *n* = 290), 327 had normal feet (56.3%), 110 pronated (18.9%), 41 hyperpronated (7.1%), 63 supinated (10.8%), and 40 hypersupinated (6.9%). Both men and women had a higher percentage of normal feet. Women had increased percentages of pronated (21.0%) and supinated (11.7%) feet than men which were 16.8% and 10.3%, respectively. The gender differences were significant (*p* value < 0.001) only in the normal, pronated, and supinated foot groups (Table 2), whereas with reference to the side of the foot, a significant difference (*p* value < 0.001) was observed in the FPI score in all foot groups (Table 3).

Table 4 summarizes the effect of group and gender differences on balance, functional mobility, and hypermobility. Both foot groups and gender showed a significant (*p* value < 0.001) effect on balance and functional mobility. However, in regard to hypermobility, the foot group (*p* value = 0.49) and gender (*p* value = 0.93) showed a nonsignificant effect on the study.

We also analyzed between-subject effects for all variables. With regard to balance, the foot group showed 81% (*ηp*^2^ = 0.816) (*p* value < 0.001), gender only showed 1.5% (*ηp*^2^ = 0.015) (*p* value = 0.004), and both foot group and gender as a whole demonstrated a 50% (*ηp*^2^ = 0.050) (*p* value < 0.001) effect on balance.

With regard to functional mobility (stair ascent), the foot group exhibited 97% (*ηp*^2^ = 0.975) (*p* value < 0.001), gender showed only 3% (*ηp*^2^ = 0.030) (*p* value < 0.001), and both foot group and gender as a whole demonstrated a 12.8% (*ηp*^2^ = 0.128) (*p* value < 0.001) effect on the stair ascent test. With reference to functional mobility (stair descent), the foot group exhibited 98% (*ηp*^2^ = 0.987) (*p* value < 0.001), gender showed 28% (*ηp*^2^ = 0.284) (*p* value < 0.001), and both foot group and gender as a whole exhibited a 20% (*ηp*^2^ = 0.204) (*p* < 0.001) effect on the stair descent test.

With regard to hypermobility, the foot group showed 49% (*ηp*^2^ = 0.493) (*p* value < 0.001), gender only showed 0.2% (*ηp*^2^ = 0.002) (*p* value = 0.33), and both foot group and gender as a whole demonstrated only a 10% (*ηp*^2^ = 0.104) (*p* value < 0.001) effect on hypermobility.

Table 5 summarizes the correlation of FPI values in different foot groups with age, height, weight, BMI, hypermobility, functional mobility, and balance. Both normal and pronated foot groups showed a very low correlation with all variables. The hyperpronated foot group demonstrated a moderate correlation with hypermobility (*r* = 0.312). The supinated foot group showed a strong correlation with functional mobility (stair ascent, *r* = 0.526; stair descent, *r* = 0.704) and a moderate correlation with balance (*r* = 0.302). The hypersupinated foot group showed a moderate correlation with BMI (*r* = 0.339). Overall, the total FPI scores in this study showed a moderate correlation with hypermobility (*r* = 0.404) and a high correlation with balance (*r* = 0.582).

## 4. Discussion

The present study established the reference values of FPI for healthy adult men and women in Saudi Arabia aged between 18 and 30 years. We found that the participant's foot posture in this study was classified as neutral with some degrees of pronation, with men demonstrating higher scores than women.

This finding was similar to previous studies carried out in Spain (2.0 ± 4.3) and Taiwan (0 ± 5.5) [28, 29]. During standing on both feet, Saudi Arabian young adults tend to present a slightly pronated foot posture, which is treated as physiological because most feet during dynamic foot functions present this posture [30].

Our study reported a higher percentage of pronated feet (46.4%) and a lower percentage of supinated feet (22.4%); these observations agree with those of previous studies [13, 30]. Such type of foot posture would be understandable considering that after weight acceptance, the foot slowly moves into pronation and attains maximum pronation in midstance. In this position, the midtarsal joint unlocks, and the foot stretches and becomes more flexible to accommodate the underlying surfaces that help to assist in maintaining balance [31].

However, the FPI score in pronated foot posture was higher in women than men due to structural changes, such as excessive ligamentous laxity, hypermobility of the joints [32], and decreased muscle strength, all of which are responsible for maintaining the height of the medial longitudinal arch that leads to pronated foot posture in women [33]. We also observed a higher percentage of supinated feet in females (18.9%) than in males (16.4%), which is not in agreement with Redmond et al. [14] or Fleiss [28]. We believe that this discrepancy is due to the different characteristics of sample size and measurement variability.

We also observed significant differences in FPI values in relation to gender in all foot groups except in the hyperpronated and hypersupinated foot groups, in contrast with previous studies [4, 29]. This observation may be because those studies are frequently limited by the range of age and unequal sample size, which may not be representative of the general population. Another important finding in this study was the variations in left foot FPI value with the right foot in different foot groups, which were similar to those reported by Cain et al. [5] and Redmond et al. [14]. The human body is asymmetric, and the left foot is more associated with weight-bearing function, whereas the right foot is more related to the forces of the body during locomotion [34]. In this sense, these variations between the right and left feet have been found to be significant in the study and support our hypothesis that side differences can be associated with FPI.

The current study found no association between BMI and FPI. Although significant differences were seen in height, weight, and BMI between men and women, the highest scores of FPI were not correlated with higher body mass. This result is consistent with that described in previous studies [14, 16]. However, one crucial finding in the present study was a moderate correlation of BMI with FPI in the hypersupinated foot group; this result may be related to the excessive overload on the plantar fascia increasing its thickness in the hypersupinated foot group. Increased thickness, in turn, increases the tensile forces on the Achilles tendon during the stance phase, as does body weight [35], and maybe the causative factor for its association. Although we found a moderate correlation, our study was not able to determine whether this relationship is the result of a higher BMI effect on plantar fascia or the overloading issue that the weight has on plantar fascia thickness.

The results of the present study demonstrated that balance has a moderate correlation with FPI in the supinated and hypersupinated foot groups, which is in contrast with previous findings [17, 21]. We also found a significant (*p* value < 0.001) size effect and interaction effect of the foot group and gender on balance in this study. However, this balance can be interrupted by either reduced afferent feedback or any small dynamic change in the structure of the feet [36]. Therefore, we believe that these minor biomechanical alterations, specifically in supinated foot postures, may affect the peripheral input through changes in joint flexibility by influencing the postural-control strategies [37], which support the findings in this study. It seems that the supinated feet may have difficulty in reacting and adjusting quickly when the body attempts to maintain balance. Hence, we conclude that the foot type should be carefully considered during clinical evaluations of balance measurements.

In terms of functional mobility, our results demonstrated that the FPI of the pronated and supinated foot groups has a moderate correlation with functional mobility. However, this result is inconsistent with that of previous studies [38, 39]. In both these foot groups, increased muscle activation of the tibialis anterior and the gastrocnemius influences the foot arch, which further affects the ankle motion and agility during stair ascent and descent tests. These foot arch alterations may also lead to structural changes and affect the distribution of load on the foot and affect functional mobility [40]. In this context, we observed a significant (*p* value < 0.001) size effect and interaction effect of the foot group and gender on functional mobility. Even a slight increase or decrease in the medial longitudinal arch decreases foot mobility [15, 41]. This inconsistency with other studies could also be due to the different physiological factors of the subjects, such as mean age, height, overweight, and BMI.

Our results also established that joint hypermobility was associated only with FPI of the hyperpronated foot group, which was similar to the previous study performed by Sanchis-Sales et al. [42]. We also found that hypermobility was more common in women than men [43]. The ratio of type I to type III collagen is reduced in the skin of women, and this ratio is associated with increased joint hypermobility and laxity of other tissues than in men in this foot group [44]. According to Handler et al., the center-of-pressure (COP) trajectory was more medial in women than in men, which increases the load on the first metatarsal and leads to increased generalized joint laxity in this foot group [45]. Similarly, a study done by Ledoux et al. determined that in the hyperpronated foot group, the mechanical advantage of the peroneus longus muscle was altered, leading to reduced eversion and plantar flexion [46]. In addition, the size effect of the foot group (*p* value = 0.49), gender (*p* value = 0.33), and interaction effect of both these (*p* value = 0.10) on hypermobility were not significant in this study because hypermobility was observed more in females and in the pronated foot group only [7]. Therefore, individuals with hyperpronated foot posture should be evaluated for joint hypermobility because this improper distribution of loads on the foot arch may increase the risk of lower extremity injuries in this foot group. Although the participants in this study are young adults who are not considered a risky group, in terms of fall of risk or other injuries, the foot posture assessment can prevent injuries to lower extremities. Therefore, the results of the present study can be generalized and used for assessment procedures emphasizing preventive rehabilitation strategies.

Post hoc power analysis was performed using the G∗POWER statistical program to evaluate the power of the study. This post hoc power analysis indicated that a sample size of 581 was sufficient to detect the reference values of FPI, with an alpha value of 0.05, and the effect size of 0.11 with Cohen's *d*, and the power is estimated 0.84 in this study.

The present study has some limitations. First, it was conducted only on healthy young adults; thus, it is unclear how such a relationship may be different for subjects with either lower extremity injury or disease. Second, our results only apply to static foot assessment using the FPI because other clinical measures with dynamic foot evaluation during walking were not considered. Third, the tests used in the study are semisubjective. In addition, more studies were recommended to overcome all these limitations.

Further research should include the establishment of reference values across various age groups with similar levels of anthropometric factors, which can significantly assist both clinicians and research teams. Recognition of various abnormal foot postures and their association with balance and mobility during the clinical examination may provide information about the subject's foot and, most likely, the nature of the problem.

## 5. Conclusion

Based on the findings, the most common foot posture in both genders was ranged from neutral to slight pronation. Significant differences were found in FPI values among men and women for all the foot groups. We also found correlations between balance and FPI in the supinated and hypersupinated foot groups, between functional mobility and FPI of the pronated and supinated foot groups, and between joint hypermobility and FPI of hyperpronated foot groups.

## Figures and Tables

**Table 1:** Anthropometric data for the overall sample.

Anthropometrics	Male (*n* = 291) Mean ± SD	Female (*n* = 290) Mean ± SD	*p* value
Age (years)	21.82 ± 1.57	21.68 ± 1.73	0.31
Height (m)	1.70 ± 0.074	1.57 ± 0.059	<0.001^∗^
Weight (kg)	74.08 ± 15.58	56.65 ± 9.16	<0.001^∗^
BMI (kg/m^2^)	25.48 ± 5.17	22.92 ± 3.58	<0.001^∗^

BMI: body mass index. ^∗^Significant difference: *p* < 0.05 level.

**Table 2:** Foot posture index (FPI) values—gender differences of the sample.

	Foot group	Gender	FPI Mean ± SD	*p* value (gender)
FPIRIGHT	Normal	Male, *n* = 178 (61.1%)	3.94 ± 1.12	<0.001^∗^
		Female, *n* = 149 (51.3%)	2.24 ± 1.42	
	Pronated	Male, *n* = 49 (16.8%)	7.12 ± 0.97	<0.001^∗^
		Female, *n* = 61 (21.0%)	8.18 ± 0.86	
	Hyperpronated	Male, *n* = 16 (5.4%)	10.75 ± 1.00	0.01^∗^
		Female, *n* = 25 (8.6%)	11.44 ± 0.70	
	Supinated	Male, *n* = 29 (10.3%)	−4.41 ± 0.730	0.40
		Female, *n* = 34 (11.7%)	−4.23 ± 0.92	
	Hypersupinated	Male, *n* = 19 (6.5%)	−10.47 ± 1.42	0.30
		Female, *n* = 21 (7.2%)	−10.85 ± 0.85	
FPILEFT	Normal	Male, *n* = 178 (61.1%)	3.50 ± 1.43	<0.001^∗^
		Female, *n* = 149 (51.3%)	1.60 ± 1.10	
	Pronated	Male, *n* = 49 (16.8%)	7.06 ± 1.16	<0.001^∗^
		Female, *n* = 61 (21.0%)	7.78 ± 0.93	
	Hyperpronated	Male, *n* = 16 (5.4%)	11.12 ± 0.80	0.06
		Female, *n* = 25 (8.6%)	10.64 ± 0.81	
	Supinated	Male, *n* = 29 (10.3%)	−3.41 ± 1.05	<0.001^∗^
		Female, *n* = 34 (11.7%)	−1.17 ± 0.45	
	Hypersupinated	Male, *n* = 19 (6.5%)	−10.73 ± 1.22	0.02^∗^
		Female, *n* = 21 (7.2%)	−10.35 ± 1.55	

FPIR: foot posture index right side; FPIL: foot posture index left side. ^∗^Significant difference: *p* < 0.05 level.

**Table 3:** Foot posture index (FPI) values—side differences of the sample.

Foot group	Side	FPI Mean ± SD	*p* value (side)
Normal (*n* = 327)	FPIR	3.16 ± 1.52	<0.001^∗^
	FPIL	2.63 ± 1.60	
Pronated (*n* = 110)	FPIR	7.70 ± 1.05	<0.001^∗^
	FPIL	7.46 ± 1.01	
Hyperpronated (*n* = 41)	FPIR	11.14 ± 0.882	<0.001^∗^
	FPIL	10.82 ± 0.833	
Supinated (*n* = 63)	FPIR	−4.31 ± 0.839	<0.001^∗^
	FPIL	−2.20 ± 1.36	
Hypersupinated (*n* = 40)	FPIR	−10.67 ± 1.16	<0.001^∗^
	FPIL	−10.40 ± 1.62	

FPI: foot posture index; FPIR: foot posture index right side; FPIL: foot posture index left side. ^∗^Significant difference: *p* < 0.05 level.

**Table 4:** Effect of groups and gender differences on balance, stair ascent, stair descent, and hypermobility.

	Normal (*n* = 327)		Pronated (*n* = 110)		Hyperpronated (*n* = 41)		Supinated (*n* = 63)		Hypersupinated (*n* = 40)		Main effect of group		Main effect of gender		Interaction effect of group and gender	
	Male (*n* = 178) (mean ± SD)	Female (*n* = 149) (mean ± SD)	Male (*n* = 49) (mean ± SD)	Female (*n* = 61) (mean ± SD)	Male (*n* = 16) (mean ± SD)	Female (*n* = 25) (mean ± SD)	Male (*n* = 29) (mean ± SD)	Female (*n* = 34) (mean ± SD)	Male (*n* = 19) (mean ± SD)	Female (*n* = 21) (mean ± SD)	*F*	*p*	*F*	*p*	*F*	*p*
Balance	8.55 ± 3.07	7.57 ± 5.08	27.89 ± 4.10	23.63 ± 3.85	8.38 ± 1.23	6.84 ± 2.16	5.40 ± 0.97	6.68 ± 1.73	5.47 ± 1.31	5.05 ± 0.60	634.11	<0.001^∗^	8.434	0.004^∗^	7.46	<0.001^∗^
Stair ascent	3.83 ± 0.063	3.86 ± 0.0955	3.54 ± 0.067	3.68 ± 0.212	3.98 ± 0.136	4.16 ± 0.111	5.46 ± 0.089	5.61 ± 0.084	6.20 ± 0.345	5.99 ± 0.143	5617.8	<0.001^∗^	17.89	<0.001^∗^	21.0	<0.001^∗^
Stair descent	3.63 ± 0.060	3.65 ± 0.064	3.32 ± 0.113	3.40 ± 0.143	3.71 ± 0.061	3.90 ± 0.103	5.20 ± 0.086	5.49 ± 0.093	5.71 ± 0.060	5.87 ± 0.114	10653.7	<0.001^∗^	226.07	<0.001^∗^	36.67	<0.001^∗^
Hypermobility	1.84 ± 1.33	2.97 ± 1.71	4.65 ± 1.54	7.29 ± 2.11	4.75 ± 1.77	7.56 ± 1.55	3.10 ± 2.52	3.58 ± 1.76	4.00 ± 1.63	4.28 ± 0.845	138.75	0.49	0.938	0.33	16.55	0.10

^∗^Significant difference: *p* < 0.05 level.

**Table 5:** Correlation of FPI values with age, height, weight, BMI, hypermobility, functional mobility, and balance.

	Age		Height		Weight		BMI		Hypermobility		Functional mobility (stair ascent)		Functional mobility (stair descent)		Balance	
Foot group	*R*	*p*	*R*	*p*	*R*	*p*	*R*	*p*	*R*	*p*	*R*	*p*	*R*	*p*	*R*	*p*
*Normal (n* = 327)																
FPIR	0.120	0.03	0.281	<0.001^∗^	0.217	<0.001^∗^	0.091	0.09	0.204	<0.001^∗^	-0.100	0.07	-0.078	0.16	0.020	0.71
FPIL	0.083	0.13	0.379	<0.001^∗^	0.272	<0.001^∗^	0.104	0.06	0.213	<0.001^∗^	-0.077	0.16	-0.060	0.28	0.113	0.04^∗^
Total FPI	0.099	0.01	0.326	<0.001^∗^	0.242	<0.001^∗^	0.097	0.01^∗^	0.206	<0.001^∗^	-0.087	0.02^∗^	-0.067	0.08	0.067	0.08
*Pronated (n* = 110)																
FPIR	-0.123	0.20	-0.363	<0.001^∗^	-0.295	0.002^∗^	-0.164	0.08	0.079	0.41	0.332	<0.001^∗^	0.199	0.03^∗^	-0.072	0.45
FPIL	0.016	0.86	-0.173	0.07	-0.221	0.02^∗^	-0.171	0.07	0.053	0.58	0.129	0.18	0.072	0.45	0.101	0.29
Total FPI	-0.052	0.44	-0.264	<0.001^∗^	-0.255	<0.001^∗^	-0.167	0.01^∗^	0.065	0.33	0.227	<0.001^∗^	0.134	0.04^∗^	0.016	0.81
*Hyperpronated (n* = 41)																
FPIR	0.065	0.68	-0.370	0.01^∗^	-0.202	0.20	-0.019	0.90	0.096	0.55	0.044	0.78	0.499	<0.001^∗^	-0.022	0.89
FPIL	-0.045	0.77	0.150	0.34	0.246	0.12	0.194	0.22	0.312	0.18	-0.331	0.03^∗^	-0.246	0.12	-0.041	0.80
Total FPI	0.011	0.92	-0.115	0.30	0.015	0.89	0.083	0.45	0.150	0.18	-0.136	0.22	0.134	0.22	-0.031	0.78
*Supinated (n* = 63)																
FPIR	0.179	0.16	-0.154	0.22	-0.094	0.46	-0.007	0.95	-0.139	0.27	0.004	0.97	-0.014	0.91	0.213	0.09
FPIL	-0.012	0.92	-0.573	<0.001^∗^	-0.582	<0.001^∗^	-0.402	0.01^∗^	-0.232	0.06	0.526	<0.001^∗^	0.704	<0.001^∗^	0.302	0.01^∗^
Total FPI	0.043	0.63	-0.294	<0.001^∗^	-0.281	<0.001^∗^	-0.179	0.04^∗^	-0.140	0.11	0.232	<0.001^∗^	0.306	<0.001^∗^	0.190	0.03^∗^
*Hypersupinated (n* = 40)																
FPIR	-0.258	0.10	0.291	0.06	-0.046	0.77	0.054	0.72	0.226	0.16	0.403	0.01^∗^	0.042	0.79	0.302	0.05
FPIL	-0.138	0.39	-0.188	0.24	0.019	0.90	0.339	0.02^∗^	0.067	0.68	-0.027	0.86	0.281	0.07	0.055	0.73
Total FPI	-0.184	0.10	0.012	0.94	-0.008	0.94	-0.017	0.88	0.131	0.24	0.149	0.18	0.178	0.11	0.155	0.16
*Total (n* = 581)																
FPIL	0.36	0.38	0.184	0.001^∗^	0.074	0.07	-0.039	0.34	0.404	<0.001^∗^	-0.555	<0.001^∗^	-0.602	<0.001^∗^	0.582	<0.001^∗^
FPIR	0.41	0.32	0.150	<0.001^∗^	0.056	0.17	-0.034	0.40	0.401	<0.001^∗^	-0.549	<0.001^∗^	-0.605	<0.001^∗^	0.568	<0.001^∗^
Overall FPI	-0.039	0.13	0.092	0.008^∗^	0.004	0.87	-0.059	0.02	0.206	<0.001^∗^	-0.827	<0.001^∗^	-0.834	<0.001^∗^	0.454	<0.001^∗^

FPIL: foot posture index left side; FPIR: foot posture index left side; ^∗^significant difference: *p* < 0.05 level.

## Data Availability

All relevant data are within the paper.

## Supplementary Materials

Supplementary materials It includes the tables of between-subject effects and pair-wise comparisons of all the outcome measures for different foot groups.
