# Supplementary material for: Foot Posture Index Reference Values among Young Adults in Saudi Arabia and Their Association with Anthropometric Determinants, Balance, Functional Mobility, and Hypermobility
Source: Biomed Res Int. 2021 Mar 28;2021:8844356. doi: 10.1155/2021/8844356 (PMC8019632; doi:10.1155/2021/8844356)
Supplement: Supplementary materials — It includes the tables of between-subject effects and pair-wise comparisons of all the outcome measures for different foot groups. [file 8844356.f1.docx]

| **Tests of Between-Subjects Effects** | | | | | | |
| --- | --- | --- | --- | --- | --- | --- |
| Dependent Variable: BALANCE | | | | | | |
| Source | Type III Sum of Squares | df | Mean Square | F | Sig. | Partial Eta Squared |
| Corrected Model | 35304.039^a^ | 9 | 3922.671 | 292.964 | .000 | .822 |
| Intercept | 35841.703 | 1 | 35841.703 | 2676.834 | .000 | .824 |
| FOOTGROUP | 33962.167 | 4 | 8490.542 | 634.115 | .000 | .816 |
| gender | 112.933 | 1 | 112.933 | 8.434 | .004 | .015 |
| FOOTGROUP * gender | 399.935 | 4 | 99.984 | 7.467 | .000 | .050 |
| Error | 7645.455 | 571 | 13.390 |  |  |  |
| Total | 121271.831 | 581 |  |  |  |  |
| Corrected Total | 42949.493 | 580 |  |  |  |  |
| a. R Squared = .822 (Adjusted R Squared = .819) | | | | | | |

| **Pairwise Comparisons** | | | | | | | | | | | | | |
| --- | --- | --- | --- | --- | --- | --- | --- | --- | --- | --- | --- | --- | --- |
| Dependent Variable: BALANCE | | | | | | | | | | | | | |
| (I) FOOTGROUP | (J) FOOTGROUP | | | Mean Difference (I-J) | | Std. Error | | Sig.^b^ | | 95% Confidence Interval for Difference^b^ | | | |
|  |  |  |  |  |  |  |  |  |  | Lower Bound | | Upper Bound | |
| NORMAL | PRONATED | | | -17.698^*^ | | .383 | | .000 | | -18.779 | | -16.617 | |
|  | HYPERPRONATED | | | .448 | | .622 | | 1.000 | | -1.304 | | 2.201 | |
|  | SUPINATED | | | 2.021^*^ | | .507 | | .001 | | .591 | | 3.451 | |
|  | HYPERSUPINATED | | | 2.797^*^ | | .616 | | .000 | | 1.062 | | 4.533 | |
| PRONATED | NORMAL | | | 17.698^*^ | | .383 | | .000 | | 16.617 | | 18.779 | |
|  | HYPERPRONATED | | | 18.146^*^ | | .668 | | .000 | | 16.263 | | 20.029 | |
|  | SUPINATED | | | 19.719^*^ | | .563 | | .000 | | 18.131 | | 21.306 | |
|  | HYPERSUPINATED | | | 20.495^*^ | | .663 | | .000 | | 18.628 | | 22.362 | |
| HYPERPRONATED | NORMAL | | | -.448 | | .622 | | 1.000 | | -2.201 | | 1.304 | |
|  | PRONATED | | | -18.146^*^ | | .668 | | .000 | | -20.029 | | -16.263 | |
|  | SUPINATED | | | 1.572 | | .746 | | .356 | | -.531 | | 3.675 | |
|  | HYPERSUPINATED | | | 2.349^*^ | | .824 | | .045 | | .027 | | 4.670 | |
| SUPINATED | NORMAL | | | -2.021^*^ | | .507 | | .001 | | -3.451 | | -.591 | |
|  | PRONATED | | | -19.719^*^ | | .563 | | .000 | | -21.306 | | -18.131 | |
|  | HYPERPRONATED | | | -1.572 | | .746 | | .356 | | -3.675 | | .531 | |
|  | HYPERSUPINATED | | | .777 | | .741 | | 1.000 | | -1.312 | | 2.865 | |
| HYPERSUPINATED | NORMAL | | | -2.797^*^ | | .616 | | .000 | | -4.533 | | -1.062 | |
|  | PRONATED | | | -20.495^*^ | | .663 | | .000 | | -22.362 | | -18.628 | |
|  | HYPERPRONATED | | | -2.349^*^ | | .824 | | .045 | | -4.670 | | -.027 | |
|  | SUPINATED | | | -.777 | | .741 | | 1.000 | | -2.865 | | 1.312 | |
| Based on estimated marginal means | | | | | | | | | | | | | |
| *. The mean difference is significant at the .05 level. | | | | | | | | | | | | | |
| b. Adjustment for multiple comparisons: Bonferroni. | | | | | | | | | | | | | |
| **Tests of Between-Subjects Effects** | | | | | | | | | | | | |  |
| Dependent Variable: STAIR ASCENT (FUNCTIONAL MOBILITY) | | | | | | | | | | | | |  |
| Source | | Type III Sum of Squares | df | | Mean Square | | F | | Sig. | | Partial Eta Squared | |  |
| Corrected Model | | 341.677^a^ | 9 | | 37.964 | | 2526.057 | | .000 | | .975 | |  |
| Intercept | | 6920.985 | 1 | | 6920.985 | | 460509.332 | | .000 | | .999 | |  |
| FOOTGROUP | | 337.719 | 4 | | 84.430 | | 5617.800 | | .000 | | .975 | |  |
| gender | | .269 | 1 | | .269 | | 17.890 | | .000 | | .030 | |  |
| FOOTGROUP * gender | | 1.263 | 4 | | .316 | | 21.005 | | .000 | | .128 | |  |
| Error | | 8.582 | 571 | | .015 | |  | |  | |  | |  |
| Total | | 10364.832 | 581 | |  | |  | |  | |  | |  |
| Corrected Total | | 350.258 | 580 | |  | |  | |  | |  | |  |
| a. R Squared = .975 (Adjusted R Squared = .975) | | | | | | | | | | | | |  |

| **Pairwise Comparisons** | | | | | | |
| --- | --- | --- | --- | --- | --- | --- |
| Dependent Variable: STAIR ASCENT (FUNCTIONAL MOBILITY) | | | | | | |
| (I) FOOTGROUP | (J) FOOTGROUP | Mean Difference (I-J) | Std. Error | Sig.^b^ | 95% Confidence Interval for Difference^b^ | |
|  |  |  |  |  | Lower Bound | Upper Bound |
| NORMAL | PRONATED | .234^*^ | .013 | .000 | .198 | .270 |
|  | HYPERPRONATED | -.226^*^ | .021 | .000 | -.285 | -.168 |
|  | SUPINATED | -1.691^*^ | .017 | .000 | -1.739 | -1.643 |
|  | HYPERSUPINATED | -2.251^*^ | .021 | .000 | -2.309 | -2.193 |
| PRONATED | NORMAL | -.234^*^ | .013 | .000 | -.270 | -.198 |
|  | HYPERPRONATED | -.460^*^ | .022 | .000 | -.524 | -.397 |
|  | SUPINATED | -1.925^*^ | .019 | .000 | -1.978 | -1.872 |
|  | HYPERSUPINATED | -2.485^*^ | .022 | .000 | -2.548 | -2.422 |
| HYPERPRONATED | NORMAL | .226^*^ | .021 | .000 | .168 | .285 |
|  | PRONATED | .460^*^ | .022 | .000 | .397 | .524 |
|  | SUPINATED | -1.465^*^ | .025 | .000 | -1.535 | -1.394 |
|  | HYPERSUPINATED | -2.025^*^ | .028 | .000 | -2.102 | -1.947 |
| SUPINATED | NORMAL | 1.691^*^ | .017 | .000 | 1.643 | 1.739 |
|  | PRONATED | 1.925^*^ | .019 | .000 | 1.872 | 1.978 |
|  | HYPERPRONATED | 1.465^*^ | .025 | .000 | 1.394 | 1.535 |
|  | HYPERSUPINATED | -.560^*^ | .025 | .000 | -.630 | -.490 |
| HYPERSUPINATED | NORMAL | 2.251^*^ | .021 | .000 | 2.193 | 2.309 |
|  | PRONATED | 2.485^*^ | .022 | .000 | 2.422 | 2.548 |
|  | HYPERPRONATED | 2.025^*^ | .028 | .000 | 1.947 | 2.102 |
|  | SUPINATED | .560^*^ | .025 | .000 | .490 | .630 |
| Based on estimated marginal means | | | | | | |
| *. The mean difference is significant at the .05 level. | | | | | | |
| b. Adjustment for multiple comparisons: Bonferroni. | | | | | | |

| **Tests of Between-Subjects Effects** | | | | | | |
| --- | --- | --- | --- | --- | --- | --- |
| Dependent Variable: STAIR DESCENT (FUNCTIONAL MOBILITY) | | | | | | |
| Source | Type III Sum of Squares | df | Mean Square | F | Sig. | Partial Eta Squared |
| Corrected Model | 337.486^a^ | 9 | 37.498 | 4825.907 | .000 | .987 |
| Intercept | 6211.550 | 1 | 6211.550 | 799403.412 | .000 | .999 |
| FOOTGROUP | 331.130 | 4 | 82.782 | 10653.791 | .000 | .987 |
| gender | 1.757 | 1 | 1.757 | 226.074 | .000 | .284 |
| FOOTGROUP * gender | 1.140 | 4 | .285 | 36.676 | .000 | .204 |
| Error | 4.437 | 571 | .008 |  |  |  |
| Total | 9312.030 | 581 |  |  |  |  |
| Corrected Total | 341.923 | 580 |  |  |  |  |
| a. R Squared = .987 (Adjusted R Squared = .987) | | | | | | |

| **Pairwise Comparisons** | | | | | | | | | | | | | |
| --- | --- | --- | --- | --- | --- | --- | --- | --- | --- | --- | --- | --- | --- |
| Dependent Variable: STAIR DESCENT (FUNCTIONAL MOBILITY) | | | | | | | | | | | | | |
| (I) FOOTGROUP | (J) FOOTGROUP | | | Mean Difference (I-J) | | Std. Error | | Sig.^b^ | | 95% Confidence Interval for Difference^b^ | | | |
|  |  |  |  |  |  |  |  |  |  | Lower Bound | | Upper Bound | |
| NORMAL | PRONATED | | | .283^*^ | | .009 | | .000 | | .257 | | .309 | |
|  | HYPERPRONATED | | | -.164^*^ | | .015 | | .000 | | -.206 | | -.121 | |
|  | SUPINATED | | | -1.701^*^ | | .012 | | .000 | | -1.735 | | -1.666 | |
|  | HYPERSUPINATED | | | -2.147^*^ | | .015 | | .000 | | -2.189 | | -2.105 | |
| PRONATED | NORMAL | | | -.283^*^ | | .009 | | .000 | | -.309 | | -.257 | |
|  | HYPERPRONATED | | | -.447^*^ | | .016 | | .000 | | -.492 | | -.402 | |
|  | SUPINATED | | | -1.984^*^ | | .014 | | .000 | | -2.022 | | -1.946 | |
|  | HYPERSUPINATED | | | -2.430^*^ | | .016 | | .000 | | -2.475 | | -2.385 | |
| HYPERPRONATED | NORMAL | | | .164^*^ | | .015 | | .000 | | .121 | | .206 | |
|  | PRONATED | | | .447^*^ | | .016 | | .000 | | .402 | | .492 | |
|  | SUPINATED | | | -1.537^*^ | | .018 | | .000 | | -1.588 | | -1.486 | |
|  | HYPERSUPINATED | | | -1.983^*^ | | .020 | | .000 | | -2.039 | | -1.927 | |
| SUPINATED | NORMAL | | | 1.701^*^ | | .012 | | .000 | | 1.666 | | 1.735 | |
|  | PRONATED | | | 1.984^*^ | | .014 | | .000 | | 1.946 | | 2.022 | |
|  | HYPERPRONATED | | | 1.537^*^ | | .018 | | .000 | | 1.486 | | 1.588 | |
|  | HYPERSUPINATED | | | -.446^*^ | | .018 | | .000 | | -.497 | | -.396 | |
| HYPERSUPINATED | NORMAL | | | 2.147^*^ | | .015 | | .000 | | 2.105 | | 2.189 | |
|  | PRONATED | | | 2.430^*^ | | .016 | | .000 | | 2.385 | | 2.475 | |
|  | HYPERPRONATED | | | 1.983^*^ | | .020 | | .000 | | 1.927 | | 2.039 | |
|  | SUPINATED | | | .446^*^ | | .018 | | .000 | | .396 | | .497 | |
| Based on estimated marginal means | | | | | | | | | | | | | |
| *. The mean difference is significant at the .05 level. | | | | | | | | | | | | | |
| b. Adjustment for multiple comparisons: Bonferroni. | | | | | | | | | | | | | |
| **Tests of Between-Subjects Effects** | | | | | | | | | | | | |  |
| Dependent Variable: BSCALE (HYPER MOBILITY) | | | | | | | | | | | | |  |
| Source | | Type III Sum of Squares | df | | Mean Square | | F | | Sig. | | Partial Eta Squared | |  |
| Corrected Model | | 1719.889^a^ | 9 | | 191.099 | | 67.857 | | .000 | | .517 | |  |
| Intercept | | 5165.957 | 1 | | 5165.957 | | 1834.361 | | .000 | | .763 | |  |
| FOOTGROUP | | 1563.058 | 4 | | 390.765 | | 138.755 | | .000 | | .493 | |  |
| gender | | 2.641 | 1 | | 2.641 | | .938 | | .333 | | .002 | |  |
| FOOTGROUP * gender | | 186.473 | 4 | | 46.618 | | 16.553 | | .000 | | .104 | |  |
| Error | | 1608.060 | 571 | | 2.816 | |  | |  | |  | |  |
| Total | | 10839.000 | 581 | |  | |  | |  | |  | |  |
| Corrected Total | | 3327.948 | 580 | |  | |  | |  | |  | |  |
| a. R Squared = .517 (Adjusted R Squared = .509) | | | | | | | | | | | | |  |

| **Pairwise Comparisons** | | | | | | |
| --- | --- | --- | --- | --- | --- | --- |
| Dependent Variable: BSCALE (HYPER MOBILITY) | | | | | | |
| (I) FOOTGROUP | (J) FOOTGROUP | Mean Difference (I-J) | Std. Error | Sig.^b^ | 95% Confidence Interval for Difference^b^ | |
|  |  |  |  |  | Lower Bound | Upper Bound |
| NORMAL | PRONATED | -4.063^*^ | .176 | .000 | -4.559 | -3.568 |
|  | HYPERPRONATED | -1.745^*^ | .285 | .000 | -2.549 | -.941 |
|  | SUPINATED | -.436 | .233 | .617 | -1.092 | .220 |
|  | HYPERSUPINATED | -1.733^*^ | .282 | .000 | -2.529 | -.937 |
| PRONATED | NORMAL | 4.063^*^ | .176 | .000 | 3.568 | 4.559 |
|  | HYPERPRONATED | 2.319^*^ | .306 | .000 | 1.455 | 3.182 |
|  | SUPINATED | 3.628^*^ | .258 | .000 | 2.900 | 4.356 |
|  | HYPERSUPINATED | 2.331^*^ | .304 | .000 | 1.475 | 3.187 |
| HYPERPRONATED | NORMAL | 1.745^*^ | .285 | .000 | .941 | 2.549 |
|  | PRONATED | -2.319^*^ | .306 | .000 | -3.182 | -1.455 |
|  | SUPINATED | 1.309^*^ | .342 | .001 | .345 | 2.274 |
|  | HYPERSUPINATED | .012 | .378 | 1.000 | -1.053 | 1.077 |
| SUPINATED | NORMAL | .436 | .233 | .617 | -.220 | 1.092 |
|  | PRONATED | -3.628^*^ | .258 | .000 | -4.356 | -2.900 |
|  | HYPERPRONATED | -1.309^*^ | .342 | .001 | -2.274 | -.345 |
|  | HYPERSUPINATED | -1.297^*^ | .340 | .002 | -2.255 | -.339 |
| HYPERSUPINATED | NORMAL | 1.733^*^ | .282 | .000 | .937 | 2.529 |
|  | PRONATED | -2.331^*^ | .304 | .000 | -3.187 | -1.475 |
|  | HYPERPRONATED | -.012 | .378 | 1.000 | -1.077 | 1.053 |
|  | SUPINATED | 1.297^*^ | .340 | .002 | .339 | 2.255 |
| Based on estimated marginal means | | | | | | |
| *. The mean difference is significant at the .05 level. | | | | | | |
| b. Adjustment for multiple comparisons: Bonferroni. | | | | | | |
